# Comparative analysis of shipboard three-component magnetometer (STCM) and proton precession magnetometer (PPM) datasets in the Australian-Antarctic Ridge

**DOI:** 10.1016/j.dib.2023.109351

**Published:** 2023-06-28

**Authors:** Hakkyum Choi, Seung-Sep Kim, Sung-Hyun Park

**Affiliations:** a Division of Earth Sciences, Korea Polar Research Institute, Incheon 21990, Republic of Korea; b Department of Geological Sciences, Chungnam National University, Daejeon 34134, Republic of Korea

**Keywords:** Geomagnetic field, Vector magnetic field, Magnetometer, Marine magnetic survey, Magnetized seafloor

## Abstract

Two different types of magnetometer, the Proton Precession Magnetometer (PPM) and the Shipboard Three-Component Magnetometer (STCM), each possess its own strengths and weaknesses in their operation. The PPM can measure the total intensity of the Earth's geomagnetic field without requiring complicated post-processing and correction. However, its operation is often limited by the condition of the sea surface. In contrast, the STCM can measure three components of the Earth's field -X, Y and Z - and is not restricted by the sea condition. However, the STCM is highly sensitive to ship's viscous magnetization, which introduces significant noise into the data quality and can lead to a loss in measured geomagnetic field. The simultaneous measurements were carried out using both types of magnetometers along the same section within the Australian-Antarctic Ridge. This region experiences strong measurements of the geomagnetic field due to its proximity to the geomagnetic South Pole. We then compared the differences between the two datasets. For each dataset, we calculated a unique linear trend and subsequently removed the discrepancy between the trends. The corrected STCM data exhibited excellent agreement with the PPM data, suggesting the potential for complementary utilization of the STCM along the PPM.

**Specifications Table**

**Table:** 

Subject	Geophysics
Specific subject area	Marine Geophysics, Marine Geomagnetism, Geomagnetic measurement of magnetized seafloor
Type of data	Graph Figure
How the data were acquired	Two different types of magnetometer were operated. A proton precession magnetometer (PPM), *SeaSpy* (Marine Magnetics Corp., Markham, On, Canada), was towed near the sea surface by the *Araon* research vessel, and a shipboard three component magnetometer (STCM), *Mag-03MS100* (Bartington Instruments Ltd., Oxford, England), was installed at a higher altitude on the research vessel. Both magnetometers measured the geomagnetic field while the *Araon* sailing at a constant speed in the direction of seafloor spreading. The acquired data were filtered using the *filter1d* program in GMT (Generic Mapping Tools) software (version 5.4.2) developed by Wessel et al. [7].
Data format	Raw Filtered
Description of data collection	Two types of magnetometers measured the Earth's geomagnetic field at one-second intervals while the research vessel was sailing at a constant speed in the direction of seafloor spreading of the Australian-Antarctic Ridge.
Data source location	Institution: Korea Polar Research Institute (KOPRI) City/Town/Region: Incheon Country: Republic of Korea Latitude and longitude for collected samples/data: [60.6°S, 156.9°E] to [63.7°S, 160.6°E] (a single survey line)
Data accessibility	Repository name: Korea Polar Data Center (KPDC) Data identification number: KOPRI-KPDC-00002192 Direct URL to data: https://doi.org/22663/KOPRI-KPDC-00002192.1
Related research article	H. Choi, S.-S. Kim, J. Dyment, R. Granot, S.-H. Park, J.K. Hong, The kinematic evolution of the Macquarie Plate: A case study for the fragmentation of oceanic lithosphere, Earth Planet. Sci. Lett. 478 (2017) 132–142. https://doi.org/10.1016/j.epsl.2017.08.035

## Value of the Data

• The 'Proton Precession Magnetometer (PPM)’ can acquire high resolution magnetic data without complicated post-processing, but its operation is limited by the sea condition. However, the 'Shipboard Three-Component Magnetometer (STCM)’ is not restricted by the sea condition although it has a weakness in data quality and post-processing. In this study, the complementary usability of the STCM was effectively shown by reducing the discrepancy between the PPM and STCM datasets.
• The PPM and STCM have different operational strengths and weaknesses. This study provides the advantage of expanding the regional coverage of geomagnetic survey by allowing researchers measuring marine geomagnetic fields to choose a magnetometer suitable for operational conditions.
• The STCM is highly sensitive to ship's viscous magnetization. As a result, data loss continues to accumulate. This study is a great example of how to correct data loss resulting from the STCM. Also, it provides insights into self-calibration of ship's magnetization by identifying environmental conditions such as ship's viscous magnetization through the implementation of ‘Figure-8 turns’.

## 1 Objective

**Fig. 1:**
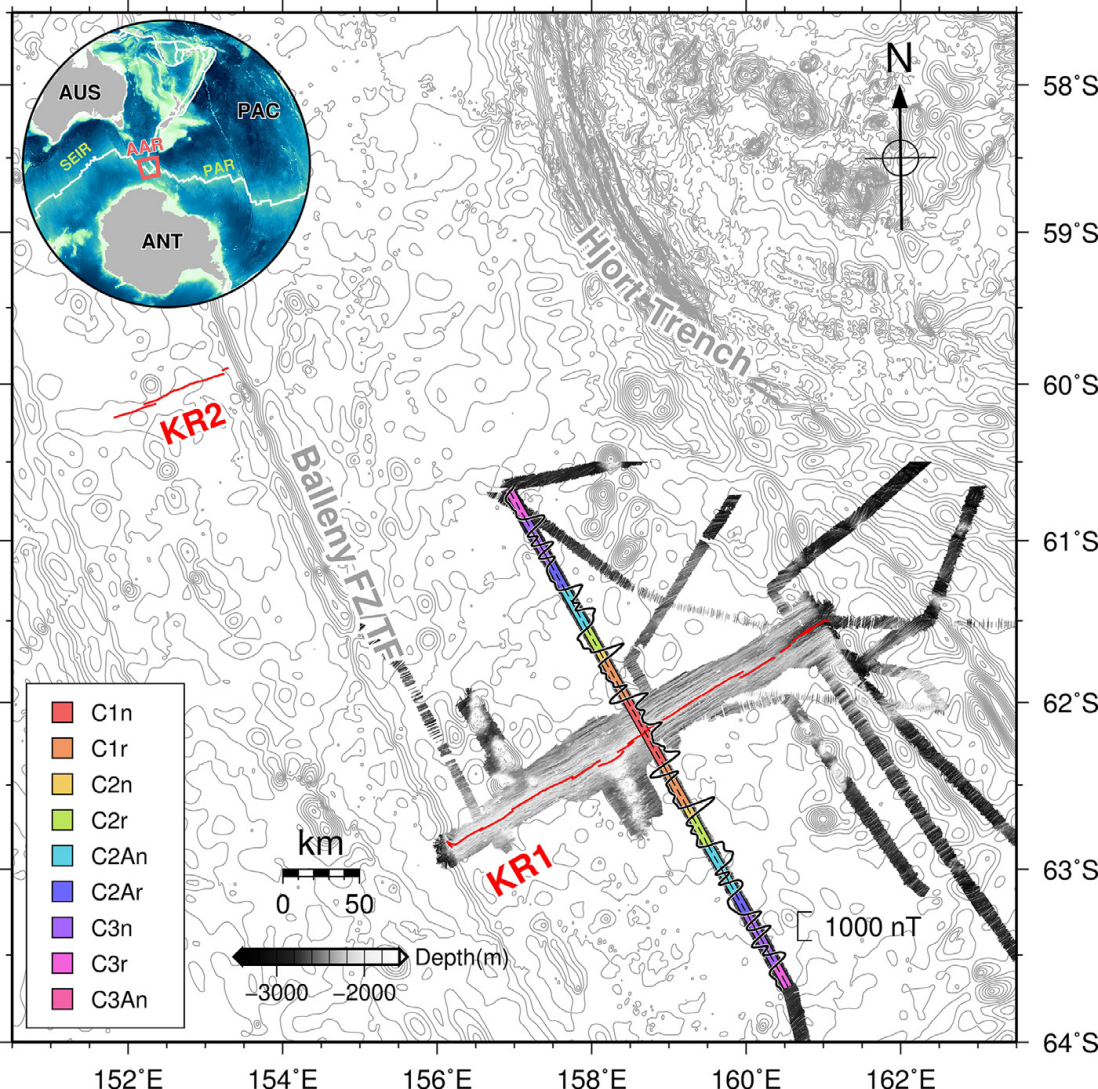
Magnetic anomalies calculated from the Proton Precession Magnetometer (PPM) data obtained at the central KR1, the easternmost segment of the Australian-Antarctic Ridge (AAR). The age of the magnetized seafloor from the magnetic anomalies is indicated by color. Data from Choi et al. [2]. Inset map shows the location of the AAR where the magnetic data was acquired. AAR: Australian-Antarctic Ridge; PAR: Pacific-Antarctic Ridge; SEIR: Southeast Indian Ridge; ANT: Antarctic Plate; AUS: Australian Plate; PAC: Pacific Plate

Over the past decade, marine geomagnetic surveys of the Australian-Antarctic Ridge (AAR) have been conducted using R/VIB *Araon* (e.g., [2,3]). The AAR represents the divergent boundary between the Australian and Antarctic plates, extending eastward from the Southeast Indian Ridge (SEIR) and converging with the Pacific-Antarctic Ridge (PAR) to the east (see inset map of Fig. 1). Due to its proximity to the geomagnetic South Pole (e.g., [6]), the seafloor generated at the ridge-axis exhibits high-intensity magnetization with symmetric spreading. To investigate the magnetized seafloor around the AAR, a series of geomagnetic data have been acquired using a Proton Precession Magnetometer (PPM) towed by the research vessel. However, in order to address the limitations of the PPM, which relies on weather conditions, the complementary use of Shipboard Three-Component Magnetometer (STCM) has been made in ongoing efforts. This study assesses the operational feasibility of the STCM by comparing the geomagnetic data obtained from both magnetometers for the exactly same section.

## 2 Data Description

Based on the precession of protons, the marine magnetometer, *SeaSpy* (Marine Magnetics Corp., Markham, ON, Canada), is capable of acquiring high-quality measurements of the Earth's magnetic field intensity. However, it has the limitation of providing only the absolute intensity of the total magnetic field. The accurate geomagnetic data obtained by *SeaSpy*, a Proton Precession Magnetometer (PPM), has proven valuable in studies investigating the kinematic evolution of tectonic plates. It allows for estimating the age of magnetized seafloor by determining the reversals in geomagnetic polarity from the Earth's magnetic field in scalar format.

The Shipboard Three-Component Magnetometer (STCM), which measures relative changes in the geomagnetic field, is significantly influenced by the ship's magnetization. This poses challenges in term of data quality and post-processing. However, the STCM offers the advantage of acquiring geomagnetic components for all three axes and is capable of collecting data irrespective of sea-surface conditions, such as sea ice in the Southern Ocean. To address the usability limitations of the PPM, we installed a portable magnetic sensor type STCM on the *Araon* research vessel, such as *Mag-03MS100* (Bartington Instruments Ltd., Oxford, England). In January 2015, both the PPM and STCM were simultaneously operated in the same section across the ridge-axis of KR1, the easternmost segment of the Australian-Antarctic Ridge (Fig. 1).

## 3 Experimental Design, Materials and Methods

The PPM and STCM data, acquired at one-second intervals, were filtered using the *filter1d* program in GMT (Generic Mapping Tools) software [7]. For the STCM data, a linear trend error accumulates due to the continuous influence of the ship's magnetization [1,4]. In other words, the discrepancy between the PPM data and STCM data increases with linear trends. To eliminate this discrepancy, a linear trend was calculated for each dataset using the *trend1d* program in GMT [7], and the difference between the two calculated trends was corrected, thus reducing the discrepancy between the PPM and STCM data.

**Fig. 2:**
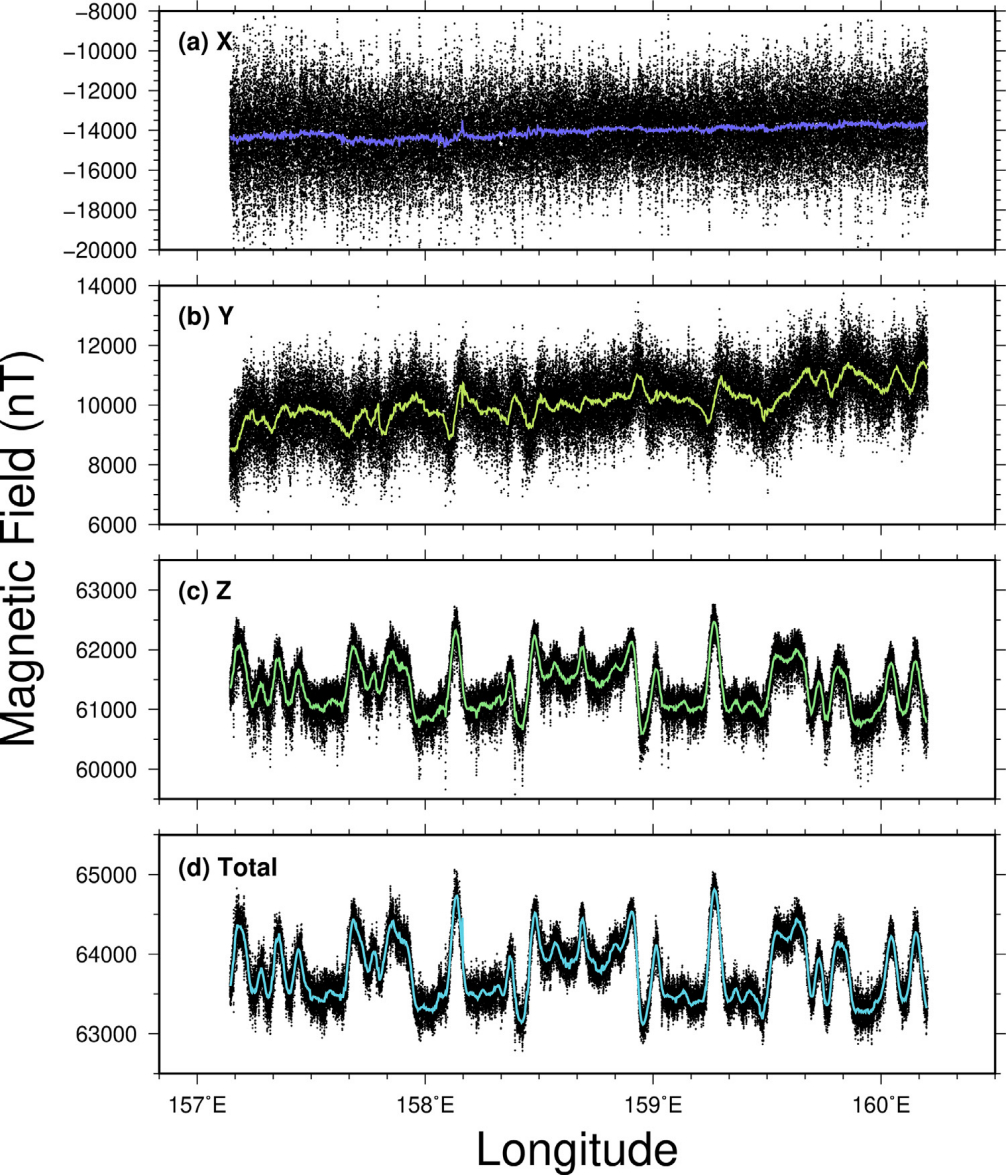
The vector magnetic field measured by Shipboard Three-Component Magnetometer (STCM) is filtered for each component. X, Y and Z refer the components north, east and downward components of the geomagnetic field. The black dots are the raw data collected, and the colored lines exhibit the filtered magnetic fields.

**Fig. 3:**
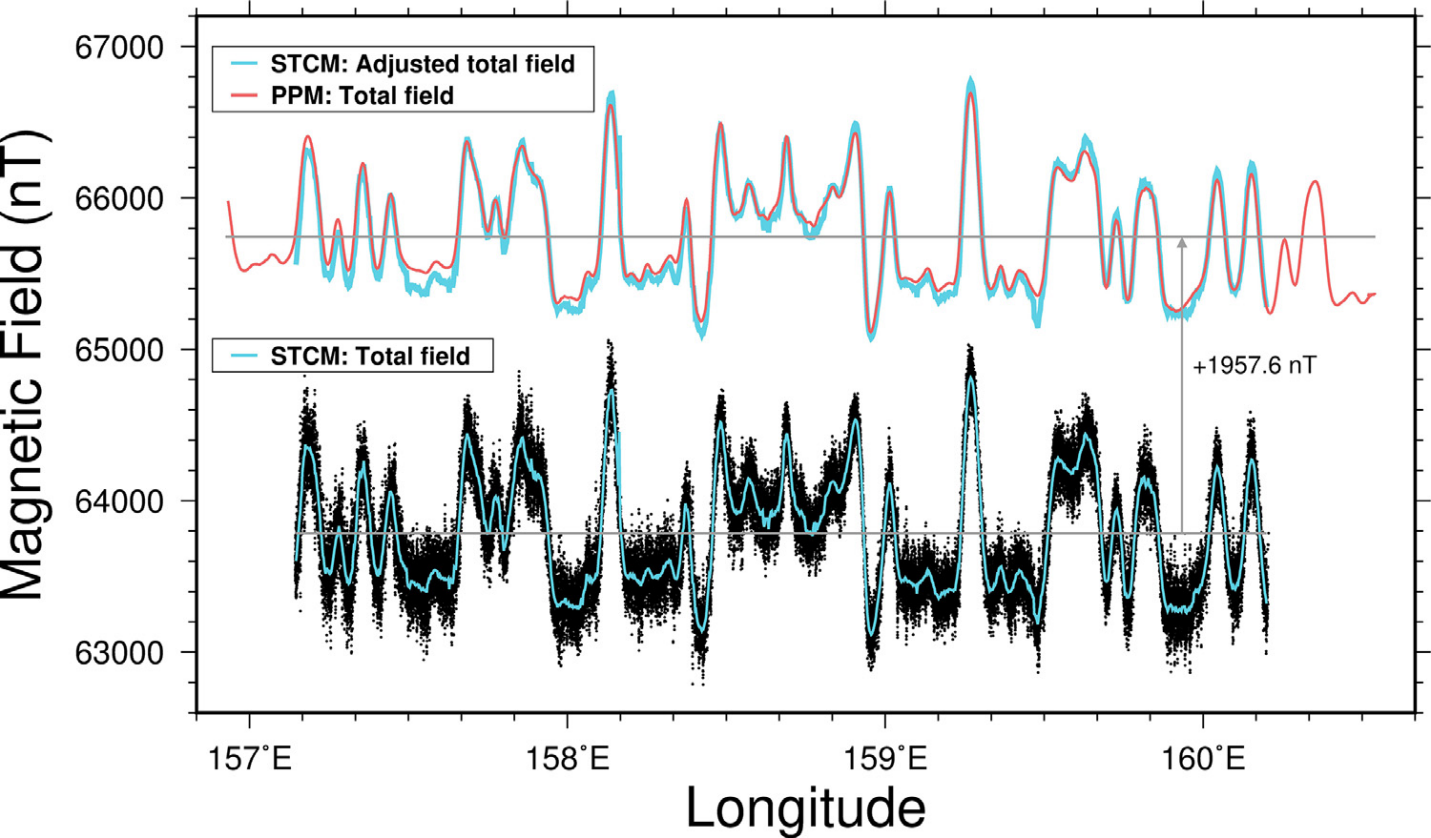
Comparison between the geomagnetic fields measured by PPM (red line) and the filtered fields measured by STCM (thin cyan line) shown in Fig. 2. Gray lines show the trends in both data.

The geomagnetic anomaly (represented by the black line) in Fig. 1 is derived from the data measured by the PPM. Utilizing the unique characteristics of the PPM, the anomaly was calculated by subtracting the International Geomagnetic Reference Field (IGRF) model 12 from the acquired and filtered magnetic data [6], without requiring a complicated post-correction process [2]. The STCM measures the vector magnetic field, specifically the north, east and downward components denoted as X, Y and Z (e.g., [5]). The STCM data was filtered for each component (Fig. 2) and then combined to calculate the total magnetic field for comparison with the PPM data (Fig. 3).

The acquired PPM and STCM showed a consistent difference of approximately 2,000 nT. In other words, the STCM data consistently exhibited a constant difference around 1,958 nT compared to the PPM data (Fig. 3), without displaying any noticeable trend of accumulating differences over time. This can be attributed to the successful implementation of ‘Figure-8 turns’ to measure the ship's viscous magnetization. It is likely that the magnetic sensor effectively self-calibrated the ship's magnetization. After correcting for this discrepancy, the STCM data aligned remarkably well with the PPM data, demonstrating a strong agreement between the two datasets (Fig. 3). These findings indicate that the considerable utility of employing the STCM in a complementary manner to the PPM.

## Ethics Statements

The datasets do not include any human subjects, animal experiments, or data collected from social media platforms.

## CRediT authorship contribution statement

**Hakkyum Choi:** Conceptualization, Methodology, Software, Formal analysis, Investigation, Writing – original draft, Visualization. **Seung-Sep Kim:** Investigation, Writing – review & editing, Supervision, Funding acquisition. **Sung-Hyun Park:** Supervision, Project administration, Funding acquisition.

## Declaration of Competing Interest

The authors declare that they have no known competing financial interests or personal relationships that could have appeared to influence the work reported in this paper.

## Data Availability

Marine Magnetic Data (PPM+STCM), central KR1 (Original data) (Korea Polar Data Center).
